# Missed diagnosis—a major barrier to patient access to obesity healthcare in the primary care setting

**DOI:** 10.1038/s41366-024-01514-6

**Published:** 2024-04-22

**Authors:** Michal Kasher Meron, Sapir Eizenstein, Tali Cukierman-Yaffe, Dan Oieru

**Affiliations:** 1 Meir Medical Center, Clalit Health Services, Kfar Saba, Israel; 2 Faculty of Medical & Health Sciences, Tel Aviv University, Tel Aviv, Israel; 3 Division of Endocrinology, Diabetes and Metabolism, Sheba Medical Center, Tel-HaShomer, Israel; 4 Maccabi Healthcare Services, Tel Aviv-Yafo, Israel

**Keywords:** Body mass index, Weight management, Preventive medicine

## Abstract

**Objective:**

To investigate whether individuals with an elevated BMI measurement, for whom a diagnosis of overweight or obesity (OW/OB) is not recorded, are less likely to be offered clinical care for obesity compared to those with a recorded diagnosis.

**Subjects:**

A retrospective cohort study using the electronic medical record database of Maccabi Healthcare Services (MHS) in Israel. Included were 200,000 adults with BMI ≥ 25 kg/m^2^ measurement recorded during a primary care visit between 2014 and 2020, and no prior diagnosis of OW/OB or related co-morbidities.

**Methods:**

The relationships between a recorded diagnosis of OW/OB and two composite outcomes: 1. A composite of referrals to screening tests for metabolic complications; 2. A composite of weight loss intervention and follow up, were analyzed using multivariate logistic regression models.

**Results:**

In only 18% of individuals, a diagnosis of OW/OB was recorded. After adjusting for multiple potential confounding factors, individuals who received a recorded diagnosis were 18% more likely to be offered an evaluation for obesity-related metabolic complication, (OR 1.18, 95% CI 1.15–1.21, *p* < 0.001), and almost twice as likely to be offered intervention and follow up for their excess body weight (OR 1.84, 95% CI 1.76–1.94, *p* < 0.001) compared to individuals with missed diagnosis. These results persisted after adjusting for inter-physician variability. In addition, male sex, older age, and Arab sector were all associated with lower rates of weight loss intervention and follow up, while young individuals were less likely to be screened for metabolic complications.

**Conclusion:**

Beyond BMI measurement, a recorded diagnosis of OW/OB is associated with statistically and clinically significant higher rates of performance of obesity care and intervention. Undiagnosed OW/OB presents a significant clinical opportunity, as recording a diagnosis of OW/OB would predict improved patient access to obesity healthcare and improved clinical outcomes.

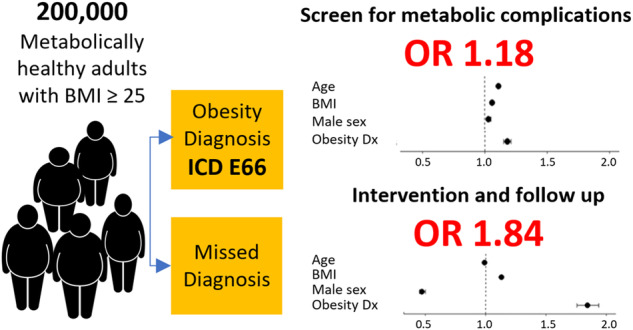

## Introduction

Overweight and obesity have been recognized by numerous health organizations worldwide as chronic medical conditions associated with increased risk of morbidity and mortality. OW/OB are states of excess fat mass defined by BMI, with overweight defined as BMI 25–29.9 kg/m² and obesity as BMI ≥ 30 kg/m² [1].

In the The American College of Cardiology (ACC), the American Heart Association (AHA) and The Obesity Society (TOS) guideline for the management of OW/OB in adults [2] as in several other published guidelines, the primary care setting is viewed as a critical entry point to the health care of people with OW/OB. The primary care physician/provider (PCP) is uniquely positioned to diagnose, inform, periodically monitor body weight, screen for obesity-related co-morbidities and offer a stepwise management plan and follow-up. It is therefore predicted that management of OW/OB by the PCP at an early stage would be associated with early detection of adiposity-related complications, facilitation of weight loss, and prevention of further weight gain.

Specifically, recording a diagnosis of OW/OB is a crucial step in treating this medical condition. Placing a formal diagnosis of OW/OB indicates recognition of OW/OB as a chronic condition by the PCP, payer, and patient. In addition, recording a diagnosis by PCP indirectly indicates that the PCP has discussed with the patient his current body weight, which has been shown to increase the patient’s motivation to implement certain lifestyle modifications [3, 4]. The medical record is a legal document therefore recording a diagnosis may increase the PCP’s and other healthcare providers’ sense of obligation to perform the related clinical workup and continued OW/OB care. Moreover, people with OW/OB often expect their PCP to raise and discuss the issue of body weight and offer them appropriate care [5]. Unfortunately, OW/OB diagnosis rates are relatively low, as less than 50% of individuals with obesity have a diagnosis of obesity recorded in their medical file [6, 7].

There is limited data indicating the beneficial effect of placing a diagnosis of OW/OB on obesity care of individuals with excess body weight in the primary care setting.

The objective of this study was to explore the relationship between the recording of OW/OB diagnosis and the performance of obesity care in the primary care setting, using the electronic medical record database of Maccabi Healthcare Services (MHS). The study cohort included adult patients with an elevated BMI of 25 or more, without prior diagnosis of OW/OB or obesity-related metabolic comorbidities. First, we assessed the relationship between recording a diagnosis of OW/OB and offering a clinical assessment and screening for obesity-related comorbidities, in accordance with published professional guidelines. Second, we investigated the association between the recording of a diagnosis of OW/OB and the offering of clinical interventions and follow up in the primary care setting.

## Research design and methods

### Study design and population

**Fig. 1:**
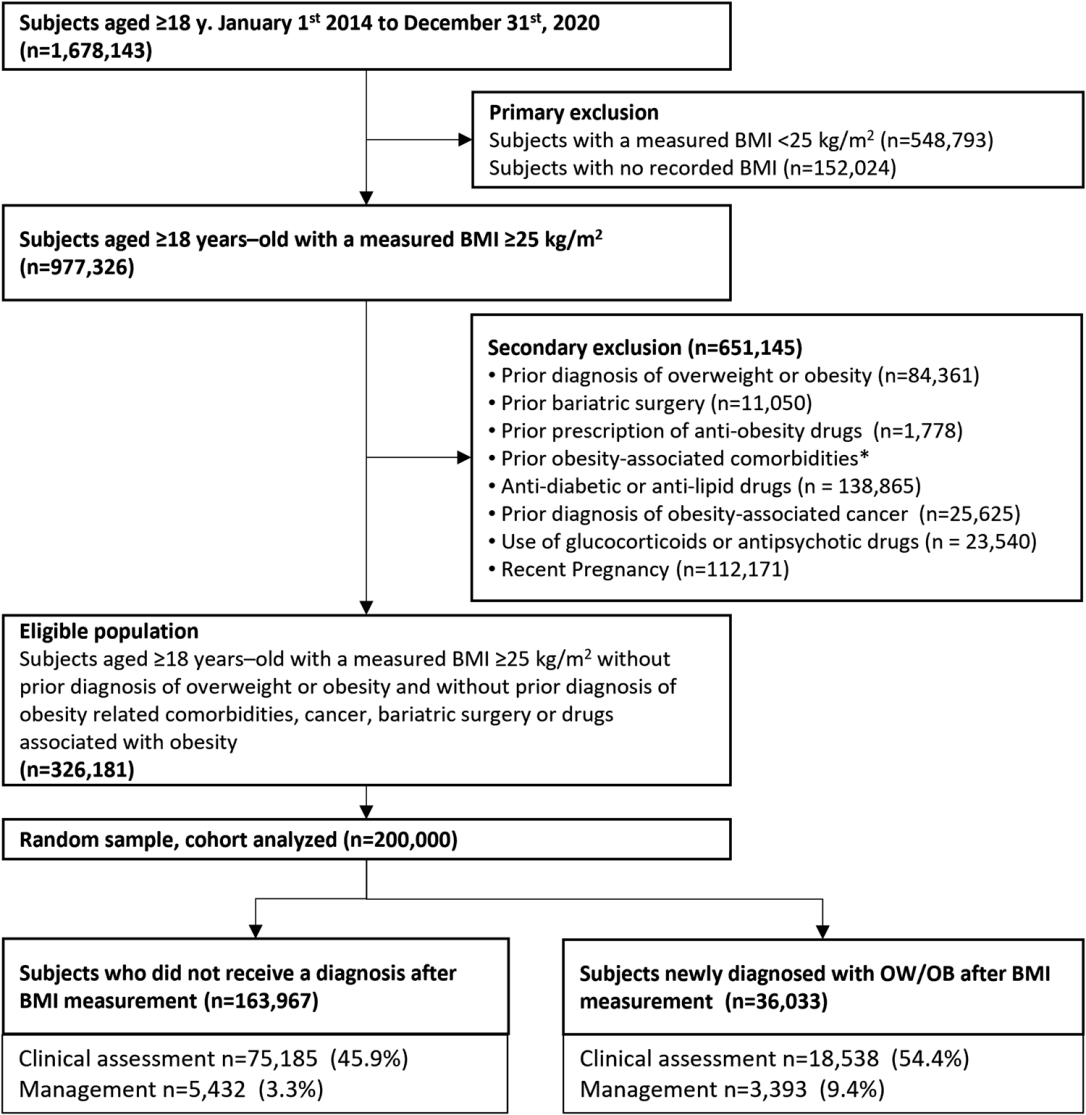
Study flow diagram. *Metabolic complications and cardiovascular disease according to MHS’ chronic disease registries including cardiovascular disease, chronic kidney disease, hypertension and diabetes mellitus, or coded diagnosis of hyperlipidemia or pre-diabetes.

The Obesity Diagnosis Study (ODiS) is a retrospective observational study using the electronic medical record database of Maccabi Healthcare Services (MHS), the second largest HMO in Israel, serving over 1.6 million adults. Study population included adults, age ≥18 years, with a BMI ≥ 25 kg/m^2^ recorded during a primary care visit (the index event). Individuals who received a recorded diagnosis of OW/OB, had bariatric surgery and/or were prescribed anti-obesity medications prior to the index event were excluded from the study. Additionally, those with prior diagnoses of obesity-related complications were excluded from the study based on any one of the following: MHS’s registries of chronic diseases including cardiovascular disease, chronic kidney disease, hypertension and diabetes mellitus (the registries’ inclusion criteria are described in supplementary material S1); Coded diagnosis of either impaired fasting glucose, hyperlipidemia, or fatty liver disease; or prior diagnosis of obesity-associated cancer, such as breast, endometrial, esophageal cancers etc. [8] according to the Israeli national cancer registry (a list of obesity related cancers is presented in supplementary material S2). We excluded individuals who were filling prescriptions within 3 months prior to the index event for oral glucocorticoid [9] or second-generation antipsychotic medications [10], which are commonly prescribed medications known to cause weight gain (Table S3). Lastly, women who delivered a child within 9 months after or 6 months before the index event were excluded. Our cohort included 326,181 eligible individuals. Using the SQL function newid(), a random sample of 200,000 people was taken as the final cohort, per the institutional review board request. Data for the index date were collected between January 1st, 2014, and December 31st, 2020. Individuals were followed up until July 2021 (Fig. 1). The Maccabi Healthcare ethics committee approved the study protocol data collection and analysis, 0036-21-MHS.

### The exposure variable

Recorded diagnosis of OW/OB was defined positive if either one of the following ICD10 codes was recorded by a PCP at or within seven days after the index event: E66, E66.0, E66.2, E66.3, E66.8, E66.9.

### Outcome variables

Clinical assessment for OW/OB-related complications was defined as a composite outcome in accordance with clinical guidelines [2] and considered positive if all of the following occurred: (I) Blood pressure (BP) was recorded within three months of the index event; (II) fasting plasma glucose (FPG) or hemoglobin A1C (HbA1c), and (III) lipid profile were ordered, within three months after the index event.

Clinical management of obesity was defined positive if (both): (I) a referral to a dietitian, a bariatric surgery consultation, or a prescription for anti-obesity drug (Table S3) was issued within 3 months after the index event; (II) a second body weight recording between 9 to 15 months after the index event, indicating clinically relevant follow up in accordance with professional guidelines [2]. Of note, we aimed to assess parameters reflecting the PCP’s adherence to clinical guidelines, rather than patient adherence, therefore, the dependent variables correspond to drug prescription and referrals issued by the PCP, rather than the actual performance of blood tests, patient attendance at dietary consultations, or drug purchases. Of note, all Israeli citizens have medical insurance covered by the state. Primary healthcare is delivered by physicians. The number of visits with the PCP is not limited, and they are usually scheduled per patient request (i.e. annual visits are not mandatory). Periodic measurement of blood pressure and body weight are strongly advocated but not mandatory or reimbursed, neither does adding a diagnosis to the patient’s medical file. Dietary consultations are covered by medical insurance, but anti-obesity medications are not.

### Covariates at baseline

Additional covariates included: sex, age, alcohol abuse, smoking status; calculated BMI, as recorded on regular clinic visits; patient sector was categorized into five categories according to their residential address at the time of the index event: Non-religious-Jewish, Jewish orthodox, Jewish-observant, Jewish-Russian immigrant, and Arab subjects; socioeconomic status (SES), based on residence at the time of the index event, coded on a 1 to 10 scale, as defined by the Israeli Bureau of Statistics [11]; Visitation to PCP during the year before the index visit; recording of an additional diagnosis at the index event other than OW/OB (i.e. reason for visit); the availability of a serum glucose test within 9 months prior to the index event; and a deidentified code assigned to each PCP at the index event.

### Statistical analysis

Baseline characteristics and potential confounders distributions, according to the presence or absence of a recorded diagnosis of OW/OB, were presented as means and standard deviations for continuous variables and as frequencies and percentages for categorical variables. Chi-square tests and independent *t* tests were performed to compare the two groups for categorical and continuous variables, respectively. Univariate analysis of the distributions of the composite outcome variables and their components according to the presence or absence of a recorded diagnosis of OW/OB was conducted. Multivariate analysis to predict each of the two predefined composite outcomes was performed by logistic regression after adjusting for potential confounders. Adjusted odds ratios with 95% confidence intervals were calculated. The goodness of fit of the model was evaluated by the Hosmer-Lemeshow test. Data were analyzed with IBM SPSS statistics software version 28.0. (SPSS Inc. Headquarters, 233 S. Wacker Drive, 11th floor Chicago, Illinois 60606, USA). Level of significance was set at 0.05 and was two-tailed.

## Results

Among 1,678,143 adult members of MHS, 977,326 had a BMI measurement of 25 or more during the study period. After excluding individuals who had a recorded diagnosis of OW/OB on their medical file before the index date, and those who had metabolic obesity-related comorbidities, or any one of the other exclusion criteria as described in methods, 326,181 people were eligible for the study, of which a random sample of 200,000 was analyzed (Fig. 1).

In this final cohort, the average age was 37.5, and average BMI was 28.8. 26% of the individuals had obesity, with BMI over 30, and 56.7% were male (Table 1). Within the study cohort only 18.0% of the subjects received a recorded diagnosis of OW/OB by their PCP at or immediately after the index event. Subjects with higher BMI were more likely to receive an OW/OB diagnosis: only 12.0% of the individuals with overweight, received a recorded diagnosis of OW/OB, compared to almost 50% of the people with obesity class III, BMI ≥ 40. People with a recorded OW/OB diagnosis were younger, more likely to be women, and had a higher BMI by 2.8 units on average, compared to people with OW/OB who did not receive a recorded diagnosis. Individuals with elevated BMI who had a recorded OW/OB diagnosis were less likely to visit their PCP during the year prior to the index event, and more likely to receive a new, non-OW/OB diagnosis at the index event, in contrast to those with missed diagnosis. In the study cohort, most subjects were non-religious Jewish adults, with less than 10% being Arab. There were minimal differences in socioeconomic status and sectorial representation between the two groups.

**Table 1:** Study cohort characteristics.

	Total population	Recorded diagnosis of OW/OB	No recorded diagnosis	*p* value
n (%)	200 000 (100)	36 033 (18.0)	163 967 (82.0)	
Age, years	37.5 ± 13.5	34.6 ± 12.6	38.1 ± 13.6	<0.001
Male gender, n (%)	113 530 (56.7)	16 298 (45.2)	97 252 (59.3)	<0.001
Weight (kg)	83 ± 13.8	88.5 ± 16.2	81.8 ± 12.9	<0.001
BMI (kg/m^2^)	28.8 ± 4.9	30.9 ± 4.4	28.1 ± 3.2	<0.001
BMI category, n(%)				
25–29.9 kg/m^2^	146 898 (73.4)	17 563 (49.0)	129 335 (79.0)	
30–34.9 kg/m^2^	39 364 (19.7)	12 328 (34.2)	27 036 (16.5)	
35–39.9 kg/m^2^	9 966 (5.0)	4 423 (12.3)	5 543 (3.4)	
40≤ kg/m^2^	3 167 (1.6)	1 603 (4.5)	1 564 (1.0)	<0.001
Alcohol, n (%)	640 (0.3)	89 (0.2)	551 (0.3)	0.007
Smoking, n (%)	35 441 (17.7)	5 818 (16.3)	29 623 (18.4)	<0.001
SES (points)	5.98 ± 2.5	5.9 ± 2.5	6.0 ± 2.5	<0.001
Sector, n (%)				
Non-religious Jewish	145 703 (72.8)	26 311 (73.0)	119 392 (72.8)	
Orthodox-Jewish	16 392 (8.2)	3 441 (9.5)	12 951 (7.9)	
Observant-Jewish	6 972 (3.5)	1 213 (3.4)	5 759 (3.5)	
Russian Immigrants	12 784 (6.4)	2 356 (6.5)	10 428 (6.4)	
Arabs	18 149 (9.1)	2 712 (7.5)	15 437 (9.4)	<0.001
Other reason for visit, n (%)*	126 791 (63.4)	23 778 (66.0)	103 013 (62.8)	<0.001
Visit within 1 year, n (%)†	98 015 (49.0)	16 232 (45.0)	81 783 (49.9)	<0.001

*Individuals who had a non-weight related diagnosis stated on their HMR on the day of BMI measurement.

†Individuals who had at least one visit with PCP prior to BMI measurement.

*SES* socioeconomic status, *OW/OB* overweight or obesity.

Overall, 56.8% of the subjects were issued referrals for fasting serum glucose levels, 48.8% were issued referrals for lipid profile blood tests, 75% of the subjects had their blood pressure taken at or immediately after the index event (Table 2). In the univariate analyses, people with OW/OB who received a recorded diagnosis of OW/OB vs those who did not, had a higher rate of the first composite outcome, 54.4% vs 45.9%. Surprisingly, individuals who received a recorded diagnosis of OW/OB were less likely to have their blood pressure taken. Overall, only 16.8% of the individuals in this cohort were issued referrals for a consultation with a nutritionist, 0.3% were issued a prescription for anti-obesity medication, 0.2% were referred to a consultation with a bariatric surgeon and 18.8% had a repeated weight measurement after a year. In the univariate analysis, individuals who had a recorded diagnosis of OW/OB had a higher rate of the second composite outcome event of obesity care, 9.4% vs. 3.3% in the control group. The event rate of each of the components of the second composite outcome was higher among individuals with a recorded diagnosis of OW/OB compared to the control group: they were more likely to be issued referrals for consultation with a nutritionist (31.0% vs. 13.7%), bariatric surgeon (0.5% vs. 0.1%), prescribed an anti-obesity medication (0.4% vs. 0.3%) or have a repeated body weight measurement after a year (26.4% vs. 17.1%).

**Table 2:** Performance rates of clinical assessment of obesity-related comorbidities and obesity management among individuals with or without recording of overweight or obesity diagnosis.

	Total population	Recorded diagnosis of OW/OB	No recorded diagnosis	*p* value
n (%)	200 000 (100)	36 033 (18)	163 967 (82)	
Issued referrals for clinical assessment within 3M after BMI measurement				
HbA1C, n (%)	34 273 (17.1)	6 931 (19.2)	27 342 (16.7)	<0.001
Fasting serum glucose, n (%)	113 646 (56.8)	22 322 (61.9)	91 324 (55.7)	<0.001
Lipid profile, n (%)	95 922 (48.8)	18 891 (52.4)	77 031 (47.0)	<0.001
Blood pressure recording, n (%)	149 902 (75.0)	25 311 (70.2)	124 591 (76.0)	<0.001
Clinical assessment Composite outcome*, n (%)	93 723 (46.9)	18 538 (54.4)	75 185 (45.9)	<0.001
Obesity management offered within 3M after BMI measurement				
Referral to a dietitian, n (%)	33 601 (16.8)	11 162 (31.0)	22 439 (13.7)	<0.001
Referral to bariatric surgery consultation, n (%)	312 (0.2)	172 (0.5)	140 (0.1)	<0.001
Prescriptions for anti-obesity pharmacotherapy, n (%)	637 (0.3)	134 (0.4)	503 (0.3)	<0.001
BMI recording within 9–15 M, n (%)	37 513 (18.8)	9 514 (26.4)	27 999 (17.1)	<0.001
Obesity management- Composite outcome†, n (%)	8 825 (4.4)	3 393 (9.4)	5 432 (3.3)	<0.001

*Composite outcome was positive if all the following were performed: referrals for serum fasting glucose or HbA1C, and lipid profile, and blood pressure recording.

†Composite outcome was positive if all the following were performed: Referring for a dietary consultation, bariatric surgery consultation or prescribing an anti-obesity drug, and repeated body weight measurement, as described in the methods section. *OW/OB* overweight or obesity. *M* months.

**Fig. 2:**
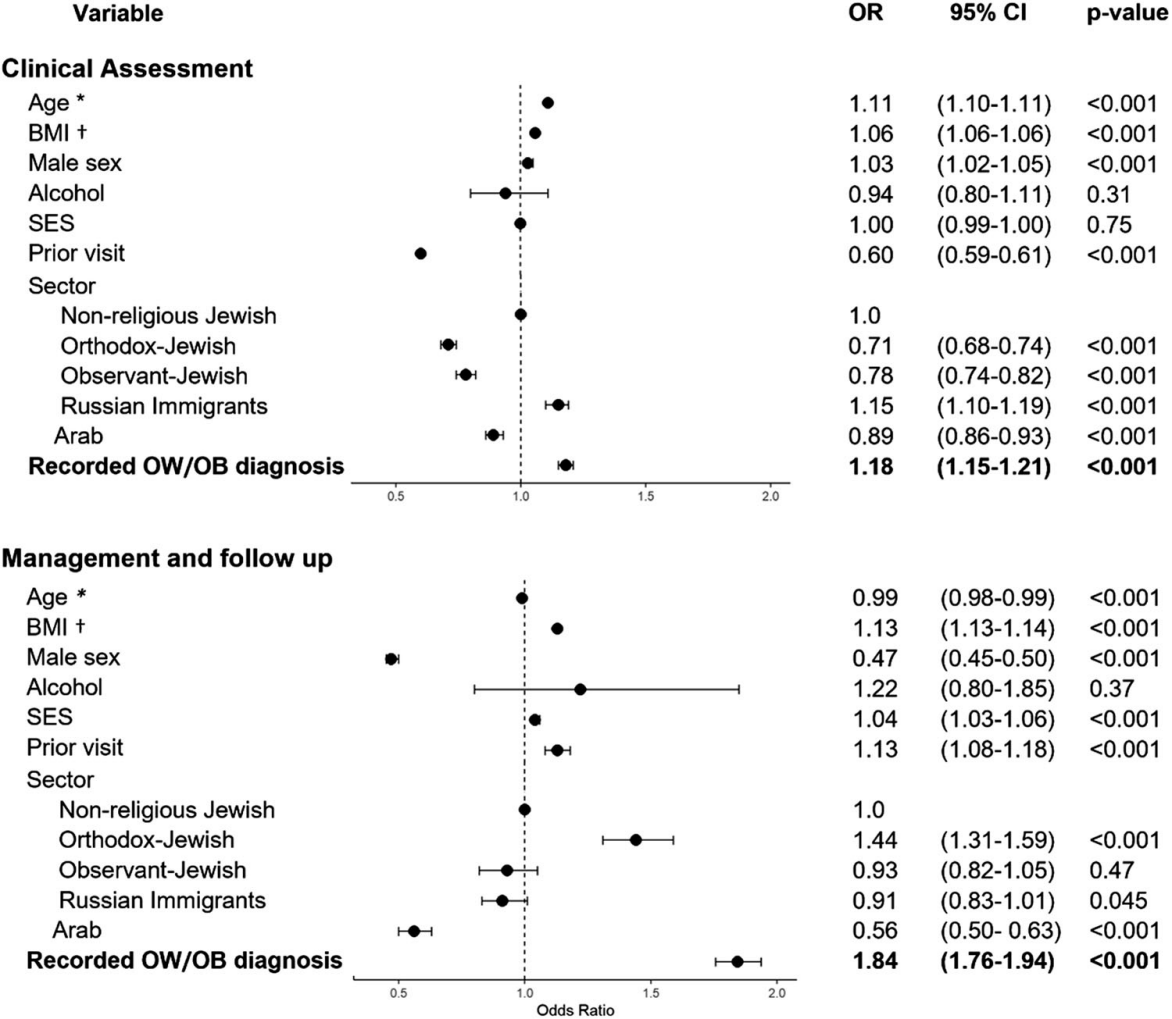
Forest plot of results of multivariate regression analyses for performance of obesity care. Performance of clinical assessment of obesity-related complications, the first composite outcome (upper panel), and performance of obesity management and follow up, the second composite outcome (lower panel). *For every 5 years increase in age; †for every 1 unit increase in BMI measured at visit. OW/OB, overweight or obesity.

After adjustment for potential confounders, there was a significant association between having a recorded diagnosis of OW/OB and the first composite outcome. In the multivariate analysis, people who were diagnosed with OW/OB by the PCP were 18% more likely to receive a clinical and biochemical evaluation for obesity-related metabolic complication, (OR 1.18, 95% CI 1.15–1.21, *p* value < 0.001). (Fig. 2, upper panel). Adjusting the model for two additional covariates, having an additional diagnosis recorded at the index event, and blood chemistry tests performed within 9 months prior to the index event, did not significantly affect this association (OR = 1.18, CI 1.15–1.21, *p* value < 0.001, table not shown). We also considered the possibility that clustering of patients within physician groups could potentially affect the study results. Therefore, we performed a sensitivity analysis with a generalized estimating equation to account for within-physician correlations, which did not affect the observed association (OR = 1.17, 95% CI 1.14–1.20, *p* value < 0.001, Table S4a). A subgroup analysis, including only individuals with obesity, BMI ≥ 30 kg/m^2^, showed similar results (OR 1.22, 95% CI 1.17–1.27, *p* value < 0.001, table not shown). According to our main model, BMI was a strong independent predictor of the first composite outcome, as with each increase of 1 unit of BMI there was a 6% increase in chances of being offered a clinical evaluation. In addition, older age, and male sex, were both associated with a higher likelihood, while having a visit with a PCP in the prior year was associated with lower likelihood of receiving a clinical assessment. Sector was another significant predictor of the composite outcome, as Orthodox- Jewish, observant-Jewish, and Arab individuals were less likely to be offered clinical and biochemical assessment compared to non-religious-Jewish individuals. The association between OW/OB diagnosis and the first composite outcome remained stable and statistically significant across different categories of age and BMI (Fig. S5).

The association between OB/OW diagnosis and the second composite outcome of obesity care was analyzed in the multivariate regression model. After adjustment for multiple potential confounders, a recorded diagnosis of OW/OB was an independent predictor of the second composite outcome, with almost twofold increase in the performance rates of clinical weight management (OR 1.84, 95% CI 1.76–1.94, *p* value < 0.001) (Fig. 2, lower panel). A subgroup analysis, including only individuals with obesity, BMI ≥ 30 kg/m^2^, showed similar results (OR 1.66, 95% CI 1.56–1.77, *p* value < 0.001, table not shown). Adjusting the model for having a non-OW/OB diagnosis recorded at the index event did not significantly affect the results (OR = 1.84, 95% CI 1.75–1.93, *p* value < 0.001, table not shown). A sensitivity analysis was performed with a generalized estimating equation to account for within-physician correlations, which did not affect the observed association between OW/OB recorded diagnosis and obesity care (OR = 1.79, CI 1.70–1.89, *p* value < 0.001, Table S4b). The association between recorded OW/OB diagnosis and the second composite outcome remained stable and statistically significant across different categories of age and BMI (Fig. S6). The main model pointed out additional predictors: male sex compared to female was associated with a 53% reduction in the likelihood of the second composite outcome. Sector was another strong independent predictor, as Arab people with OW/OB were 44% less likely whereas Orthodox- Jewish people were 44% more likely to be offered clinical care and follow up for their excess body weight compared to the reference group of non-religious Jewish individuals. Lastly, younger age and higher BMI were both independent positive predictors for the second composite outcome.

## Discussion

In this retrospective analysis of 200,000 individuals with excess body weight, those receiving a new recorded diagnosis of OW/OB were 18% more likely to be referred for screening tests for obesity-related metabolic complications, and almost twice as likely to be offered weight loss intervention and follow up compared to individuals who did not receive a recorded diagnosis. These results were highly significant and persisted also after adjustment for multiple potential confounders. In addition, male sex, older age, and Arab sector were all associated with lower rates of weight loss intervention and follow up, while young individuals were less likely to be screened for metabolic complications.

To the best of our knowledge, the current study is the first to show an association between the recording of OW/OB diagnosis and higher performance rates of clinical assessment of OW/OB- associated comorbidities. Compared to individuals with normal weight, people with obesity are at a higher risk for developing multi-morbidities across the BMI range even at the age of 30 [12]. Failure to timely diagnose OW/OB and screen for associated metabolic complications is a public health concern. Rapid weight gain is common among young adults [13], and is associated with increased risk for metabolic complications [14–16]. There are very limited data on the association between recorded diagnosis of OW/OB and performance of obesity care by medical staff. In a single-center retrospective report by Bardia et al., diagnosis of obesity was a strong predictor of formulation of an obesity treatment plan (OR = 2.39) [17]. In another small study by Banerjee et al., adding a diagnosis of obesity to the patient’s problem list was associated with increased likelihood of providers addressing obesity at future visits [18].

In this cohort of Israeli population with excess body weight, we report a relatively low rate of OW/OB diagnosis recording at or immediately after measuring an elevated BMI in alignment with previous studies, reporting low diagnosis rates especially among men [6, 17, 19, 20]. Recording a diagnosis may encourage the PCP and other healthcare providers to be proactive and offer additional workup and continued care.

This has been demonstrated in other chronic diseases. In a study by Gopalan et al. patients with type 2 diabetes, who received an ICD coded diagnosis of diabetes vs those who did not, were more likely to receive screening for vascular complications, foot care, and anti-diabetic pharmacotherapy [21]. According to ACC/TOS guidelines, screening for obesity-related comorbidities should be repeated annually, yet other professional guidelines suggest lower frequency of screening [22, 23]. In this study, adjusting the model for the availability of laboratory tests within 9 months prior to the index event did not affect the analysis result. We therefore conclude that higher rates of referrals to metabolic screening among patients with a recorded diagnosis of OW/OB cannot be attributed to lower availability of prior blood work. More than 60% of the subjects had a non-OW/OB diagnosis placed during the index event, which might suggest that the reason for most visits was non-weight-related and could partially explain the low rates that obesity care was offered. However, adjusting for this covariate did not considerably change the association. In this cohort, those who received a recorded diagnosis of OW/OB were less likely to have a visit with a PCP in previous year compared to the control group, despite having a higher BMI. This finding is somewhat surprising as previous studies reported BMI as a predictor of increased utilization of healthcare services, an association which was mostly driven by higher prevalence of chronic morbidity among people with elevated BMI [24, 25]. There are several possible explanations for this observation: Our cohort included only patients who did not have obesity-related comorbidities, and had a relatively low representation of people with severe obesity, class II and III; Patients who received a diagnosis were younger than the control group; Lastly, people with higher BMI could be prone to weight bias, which may negatively affect healthcare utilization, and postpone visits at the primary care clinic [26]. Their elevated BMI and lower healthcare utilization rates could also explain why those who received OW/OB diagnosis were more likely to have another diagnosis recorded at the index event compared to the control group. The reasons for primary healthcare utilization among patients with elevated BMI are beyond the scope of this work. Only 4.4% of the individuals were offered clinical care and follow-up in accordance with published guidelines. These low rates could be related to PCPs’ perceptions regarding their role in obesity care [27], and the reported ineffectiveness of obesity control efforts in the primary care setting [28, 29], which may lead to failure to recognize OW/OB and offer relevant clinical care. One could argue that recording a diagnosis is not by itself a trigger for management, and simply reflects the physician’s own bias, as some physicians are more interested than others in addressing obesity in their daily practice [30]. In the multivariate regression models, we noticed a differential effect of sex on the first and second outcomes: Male patients were more likely to receive clinical evaluation for obesity-related metabolic comorbidities, while female patients were more likely to have the second outcome of being offered weight loss intervention and follow up. These findings are consistent with other publications reporting higher rates of screening for cardiovascular risk factors in male patients [31, 32], while women are more likely to have their body weight measured [33] and to receive a prescription for anti-obesity drugs or referral to dietary consultation from their primary care provider [32, 34]. These sex differences could be related to physician bias as well as patient concern.

Adjusting our model for the PCP’s de-identified code allowed us to indirectly account for the inter-physician variability and did not affect the study results. To the best of our knowledge this is the first study to show the importance of recorded diagnosis irrespective of physician characteristics, in the management and care of people with OW/OB.

Ciemins et al. reported in a retrospective study that the recording of obesity diagnosis for patients with BMI of 30 or more was an independent predictor of achieving 5% weight loss after 1 year [6]. In their study, prescribing an anti-obesity medication, which reflects one of the aspects of obesity care offered by the physician, was a strong predictor of weight loss. After adjusting for anti-obesity drug prescription, obesity diagnosis was associated with a 30% increase in the likelihood of achieving a 5% weight loss or more. Their report suggests that both recorded diagnosis and physician performance increase the likelihood of weight loss. Taken together with the results of the current study, improving rates of OW/OB diagnosis recording in the primary care setting, for example by implementing automated dashboard notifications when elevated BMI is recorded [35], could increase PCP’s involvement and active role in obesity care and potentially lead to improved weight loss and metabolic outcomes.

Our study has several limitations. The analysis did not include data on free-text clinical notes, and therefore it is possible that OW/OB were verbally diagnosed or discussed during the visit, without placing a coded diagnosis, which might have led to underestimation of the association between OW/OB diagnosis and performance of obesity care by the PCP. Whether the concern of excess weight at the index event was raised by patient or physician, is a possible confounder, which this analysis did not account for. Of note, the study was conducted before the introduction and regulatory approval of new potent pharmacological interventions for obesity in clinical practice. It is possible that in coming years as effective pharmacological interventions will become widely accepted by both patient, PCP and provider, there will be an increase in the rate of OW/OB diagnosis and medical care. The goal of obesity care is to achieve weight loss and reduce future obesity related morbidity. The question whether a formal diagnosis of OW/OB by itself or performance of obesity care by the PCP leads to actual patient weight loss or prevention of obesity related complications, was not addressed in the current study, and will be the focus of our future work. Lastly, the study represents parameters of physician performance at the primary care setting in Israel, and therefore may not apply to other primary care settings around the world.

In conclusion, our study presents a strong association between the recording of OW/OB diagnosis and the offering of clinical screening and especially management for people with OW/OB in the primary care setting. Taken together, the high rates of undiagnosed OW/OB present a significant clinical opportunity, as recording a diagnosis of OW/OB would predict higher PCP’s engagement and obligation to offer obesity clinical care. Whether a recorded diagnosis of OW/OB is associated with weight control and prevention of OW/OB related complications warrants further investigation and will be the focus of our next studies.

### Supplementary information

Supplemental Material

## Data Availability

In accordance with the Israeli Ministry of Health regulations, individual-level data cannot be shared openly. Specific requests for remote access to de-identified community-level data should be directed to KSM, Maccabi Healthcare Services Research and Innovation Center.
