# Supplementary material for: Missed diagnosis—a major barrier to patient access to obesity healthcare in the primary care setting
Source: Int J Obes (Lond). 2024 Apr 22;48(7):1003–10. doi: 10.1038/s41366-024-01514-6 (PMC11216998; doi:10.1038/s41366-024-01514-6)
Supplement: Supplementary file 1 — Supplemental Material [file 41366_2024_1514_MOESM1_ESM.docx]

**Supplemental Materials**

**Supplemental material 1**

**Maccabi Healthcare Services registries**

**Diabetes mellitus registry** (1–3)**:**

Inclusion in the diabetes registry required at least one of the following criteria:

HbA1c ≥7.25%

Glucose ≥200 mg/dl (for individuals that are included in the register on this criterion alone, supporting data for the diagnosis of diabetes is required at 6 months. If none are found, the patient is deleted from the register)

Purchase of diabetes medication (supplement 3) twice in the previous 2 months

A diagnosis of diabetes (ICD-9 code) in the chart and HbA1c ≥6.5% or glucose >125 mg/dl.

These criteria have been validated and give a specificity of 99.9%.

**Cardiovascular registry** (4)**:**

Disease categories included in the cardiovascular registry:

Ischemic Heart Disease (IHD)

Congestive Heart Failure (CHF)

Peripheral Vascular Disease (PVD)

Cerebrovascular Disease (CVD)

Atrial Fibrillation (AF)

Cerebrovascular Atherosclerosis

Other disease (valve disease, cardiomyopathies, rhythm disorders)

Inclusion in the CVD registry required one of the following criteria:

Diagnosis of disease by physician (primary, cardiologist, hospitals) based on ICD-9 codes

Treatment/ procedure, e.g., CABG or PCI, based on CPT code

The electronic registry includes all patients that have been diagnosed twice or more by hospital or outpatient cardiologists, primary physicians, or pediatricians with at least one of the following clinical diagnoses, classified according to the International Classification of Diseases, Ninth revision (ICD-9) codes:

Ischemic Heart Disease (IHD) – MI (410.x,412,429.7,429.79)

Non-MI (36.x, 411.x, 414.x,429.2)

Congestive heart failure (404.x, 428.x)

Peripheral vascular disease (440.x, 441.x, 442.x, 443.x)

Cerebrovascular disease CVA (433.x1, 438.x)

TIA (435.x)

Cerebrovascular atherosclerosis (434.x0)

Atrial fibrillation (427.3x).

Patients with prior coronary artery bypass grafting (CABG) or percutaneous coronary intervention (PCI) based on relevant Current Procedural Terminology (CPT) codes (33510, 33999, 92980, 92982, 92984, 33207) were also included in the registry.

Primary acute myocardial infarction cases were identified using ICD-9 code (410.9) from hospital discharge abstracts.

To identify patients with missing hospital diagnoses, we also included patients with first AMI diagnosis from community physicians if they were hospitalized for at least 3 days during a 30-day period prior to diagnosis.

To increase the registry's specificity, patients who were diagnosed as suffering from CVD by primary physician or general hospitals prior to 2000 with no further indication were excluded from the registry.

**Chronic kidney disease (CKD) registry** (5,6)**:**

Inclusion in the CKD registry required one of the following criteria:

Pathologic glomerular filtration rate (GFR)

Proteinuria (for early stage)

Dialysis

Kidney transplant

GFR Staging:

Stage A GFR>90 and proteinuria

Stage A1 GFR 60-89 and proteinuria

Stage B GFR 30-59

Stage C GFR 15-29

Stage D GFR<15 or dialysis

**Hypertension registry** (7)**:**

Inclusion in the hypertension registry required one of the following criteria:

At least 2 physician's diagnoses or hospital records and at least 2 blood pressure readings of 140/90 mmHg or higher

For cases with abnormal measurement, but no diagnoses, 4 documented measurements are required, where at least 50% of the measurements are systolic>160 mmHg or diastolic>90mmHg

Patients with 6 or more dispensed medications for hypertension

**Supplemental Material S2**

**Obesity-associated Malignancies**

Cancers associated with overweight and obesity (8), according to the Israeli national cancer registry (INCR):

Esophageal

Gastric

Small bowel

Colon and rectum

Pancreas

Gallbladder

Liver

Endometrium

Breast

Ovary

Thyroid

Multiple myeloma

Kidney

**Table S3**

**List of Medications**

| Anti-obesity medications * | Phentermine  Liraglutide 3.0 mg  Orlistat  Lorcaserin |
| --- | --- |
| Anti-psychotic medications (9) | Risperidone  Quetiapine  Chlorpromazine  Clozapine  Olanzapine  Aripiprazole  Ziprasidone  Sertindole  Paliperidone |

*Drugs approved by the Israeli Ministry of health for the treatment of obesity during the study period 2005-2020. Sibutramine and rimonabant were discontinued in Israel before 2010 and were not included in this analysis.

**Table S4a**

Multivariate regression analysis of the first composite outcome adjusted for inter physician variability based on physician’s de-identified code

| First composite outcome |  |  |  |
| --- | --- | --- | --- |
|  | OR | 95% CI | p-value |
| **OW/OB recorded diagnosis** | **1.169** | **1.139-1.201** | **<0.001** |
| Age, per 5y | 0.908 | 0.904-0.911 | <0.001 |
| Sex | 1.046 | 1.025-1.067 | <0.001 |
| BMI at index | 0.945 | 0.942-0.948 | <0.001 |
| Alcohol abuse | 0.937 | 0.788-1.114 | 0.463 |
| Sector |  |  |  |
| 0 = Jewish, non-religious | 1 |  |  |
| 1 = Jewish, orthodox | 0.698 | 0.653-0.745 | <0.001 |
| 2 = Arab | 0.892 | 0.835-0.952 | <0.001 |
| 3 = Jewish, Russian Immigrants | 1.109 | 1.037-1.187 | 0.003 |
| 4 = Jewish, Observant | 0.785 | 0.744-0.829 | <0.001 |
| Physician | 0.999 | 0.994-1.005 | 0.814 |
| PCP visit 1y before index | 1.641 | 1.609-1.674 | <0.001 |

**Table S4b**

Multivariate regression analysis of the second composite outcome adjusted for inter physician variability based on physician’s de-identified code

|  | OR | 95% CI | p-value |
| --- | --- | --- | --- |
| **OW/OB recorded diagnosis** | **1.794** | **1.703-1.891** | **<0.001** |
| Age, per 5y | 1.016 | 1.007-1.025 | <0.001 |
| Sex | 0.469 | 0.447-0.493 | <0.001 |
| BMI at index | 0.883 | 0.879-0.887 | <0.001 |
| Alcohol abuse | 1.243 | 0.804-1.923 | 0.328 |
| Sector |  |  |  |
| 0 = Jewish, non-religious | 1 |  |  |
| 1 = Jewish, orthodox | 1.677 | 1.415-1.987 | <0.001 |
| 2 = Arab | 0.642 | 0.549-0.751 | <0.001 |
| 3 = Jewish, Russian Immigrants | 0.917 | 0.804-1.046 | 0.197 |
| 4 = Jewish, Observant | 0.995 | 0.847-1.169 | 0.951 |
| Physician | 0.962 | 0.949-0.975 | <0.001 |
| PCP visit 1y before index | 0.901 | 0.860-0.944 | <0.001 |

**Figure S5**

**A**

**B**

The association between OW/OB diagnosis and the composite outcome of clinical assessment of obesity related-complications across different categories of age (A). The association between OW/OB diagnosis and the composite outcome of clinical assessment of obesity related complications across different categories of BMI (B). OW/OB, overweight or obesity; dx, diagnosis.

**Supplemental figure S6**

**A**

**B**

The association between OW/OB diagnosis and the composite outcome of clinical management across different categories of age (A). The association between OW/OB diagnosis and the composite outcome of clinical management across different categories of BMI (B). OW/OB, overweight or obesity; dx, diagnosis.

**References**

1. Chodick G, Heymann AD, Shalev V, Kookia E. The epidemiology of diabetes in a large Israeli HMO. Eur J Epidemiol. 2003;18(12):1143–6.
2. Sella T, Shoshan A, Goren I, Shalev V, Blumenfeld O, Laron Z, et al. A retrospective study of the incidence of diagnosed Type 1 diabetes among children and adolescents in a large health organization in Israel, 2000-2008. Diabet Med. 2011 Jan;28(1):48–53.
3. Heymann AD, Chodick G, Halkin H, Kokia E, Shalev V. [Description of a diabetes disease register extracted from a central database]. Harefuah. 2007 Jan;146(1):15-17,79.
4. Shalev V, Chodick G, Goren I, Silber H, Kokia E, Heymann AD. The use of an automated patient registry to manage and monitor cardiovascular conditions and related outcomes in a large health organization. Int J Cardiol. 2011 Nov;152(3):345–9.
5. Coresh J, Turin TC, Matsushita K, Sang Y, Ballew SH, Appel LJ, et al. Decline in estimated glomerular filtration rate and subsequent risk of end-stage renal disease and mortality. JAMA. 2014 Jun;311(24):2518–31.
6. Yu J, Goldshtein I, Shalev V, Chodick G, Ish-Shalom S, Sharon O, et al. Renal impairment among postmenopausal women with osteoporosis from a large health plan in Israel. Arch Osteoporos. 2015;10:210.
7. Weitzman D, Chodick G, Shalev V, Grossman C, Grossman E. Prevalence and factors associated with resistant hypertension in a large health maintenance organization in Israel. Hypertens (Dallas, Tex 1979). 2014 Sep;64(3):501–7.
8. Flegal KM, Kruszon-Moran D, Carroll MD, Fryar CD, Ogden CL. Trends in Obesity Among Adults in the United States, 2005 to 2014. JAMA. 2016 Jun;315(21):2284–91.
9. Blumenthal SR, Castro VM, Clements CC, Rosenfield HR, Murphy SN, Fava M, et al. An electronic health records study of long-term weight gain following antidepressant use. JAMA psychiatry. 2014 Aug;71(8):889–96.
